# Real-world outcomes of elexacaftor/tezacaftor/ivacaftor in a cohort of children and adolescents with cystic fibrosis in Brazil

**DOI:** 10.36416/1806-3756/e20260204

**Published:** 2026-11-09

**Authors:** Renata Wrobel Folescu Cohen, Paloma Fernandes Coelho, Leonardo Yllel Rocha, Julia Vieira da Cunha Moreira, Natália Barboza, Katty Anne Carvalho Marins, Viviane Mauro Correa Meyer, Patrícia Fernandes Barreto Machado Costa, Tania Wrobel Folescu

**Affiliations:** 1. Instituto Fernandes Figueira/Fundação Oswaldo Cruz - IFF/Fiocruz - Rio de Janeiro (RJ) Brasil.; 2. Universidade do Estado do Rio de Janeiro - UERJ - Rio de Janeiro (RJ) Brasil.

**Keywords:** Cystic fibrosis, Cystic fibrosis transmembrane conductance regulator, Genotype

## Abstract

**Objective:**

To describe real-world clinical outcomes associated with elexacaftor/tezacaftor/ivacaftor (ETI) in people with cystic fibrosis (pwCF) followed at a pediatric referral center in Brazil.

**Methods:**

This was a retrospective analysis nested within a prospective cohort study conducted between February of 2023 and February of 2026. We included individuals who had confirmed cystic fibrosis; who had initiated treatment with ETI; and who were followed for at least six months. Outcomes assessed included BMI-for-age z-score (BAZ), percent predicted FEV_1_, sweat chloride concentration (SCC), microbiological data, and adverse events. Data were collected from baseline prior to ETI initiation and from follow-up visits at 6-12 months after initiation of ETI treatment.

**Results:**

A total of 108 pwCF (males, 50.9%; mean age, 10.2 ± 3.7 years) were included. Genotypes were F/F (46.3%), F/non-F (47.2%), and non-F/non-F (6.5%). ETI was associated with significant improvements in BAZ and FEV_1_ percentage points (ppFEV_1_), with a mean individual increase of 16.9 percentage points in lung function. Median SCC decreased from 95 mmol/L to 41 mmol/L, with most pwCF falling below the diagnostic threshold. No significant correlation was observed between SCC reduction and changes in BAZ or ppFEV_1_. The proportion of individuals with at least one positive respiratory culture for Pseudomonas aeruginosa declined from 54.63% before ETI to 23.58% within 6-12 months of treatment (p < 0.0001). Adverse events were mild: 5.6% developed rash requiring temporary interruption; two had elevated creatine phosphokinase levels; and no clinically significant liver enzyme elevation or permanent discontinuation occurred.

**Conclusions:**

ETI was effective and well tolerated, leading to meaningful improvements in nutritional status, lung function, and cystic fibrosis transmembrane conductance regulator restoration, as well as to reduced P. aeruginosa detection, in this real-world pediatric cohort.

## INTRODUCTION

Cystic fibrosis is a life-limiting autosomal recessive disease caused by pathogenic variants in the gene encoding the cystic fibrosis transmembrane conductance regulator (CFTR) protein. CFTR dysfunction at the epithelial surface leads to defective chloride and bicarbonate transport, resulting in viscous secretions, progressive bronchiectasis, exocrine pancreatic insufficiency, and multiorgan complications. Until recently, therapeutic strategies focused exclusively on downstream consequences of CFTR dysfunction rather than on its molecular cause.^1^

The advent of CFTR modulators has fundamentally altered this paradigm. These small molecules target the defective protein directly: potentiators enhance channel-open probability at the cell surface, while correctors improve protein folding and trafficking to the membrane. The triple combination of elexacaftor, tezacaftor, and ivacaftor (ETI)-comprising two correctors and one potentiator-constitutes the most effective CFTR modulator regimen to date and is widely referred to as highly effective modulator therapy (HEMT).^1^

Phase III randomized controlled trials have demonstrated that ETI produces substantial and sustained improvements in predicted FEV_1_, BMI, sweat chloride concentration (SCC), and health-related quality of life across multiple genotypes, with concomitant reductions in pulmonary exacerbation rates and hospitalizations.^2^ ^-^ ^4^ Post-approval registry analyses from North America, Europe, and Australia have corroborated these benefits under routine clinical conditions and across previously underrepresented subgroups, including young children, adolescents, and people with cystic fibrosis (pwCF) with rare or minimal-function variants.^1^

Despite this robust global evidence base, published real-world experience from Latin America remains limited.^5^ Brazil carries one of the largest population of pwCF in the region, with over 6,400 individuals registered in the national cystic fibrosis registry as of 2021 and a population that remains predominantly pediatric-with a median age of approximately 10 years and more than 74% of pwCF being < 18 years of age.^6^ Brazil has a substantial genetic diversity among affected individuals, reflecting the country’s admixed ethnic composition. This differs markedly from European and North American cohorts, with a lower frequency of the F508del homozygous genotype and a broader representation of rare and population-specific variants. F508del is present in only 44.4% of CFTR alleles among Brazilian pwCF-compared with approximately 70% in European registries-with 331 distinct CFTR variants identified nationally, underscoring the breadth of genetic diversity.^3^ ^,^ ^6^ ^,^ ^7^ ETI was incorporated into Brazil’s public health system, the *Sistema Único de Saúde* (SUS, Unified Health Care System) in 2023 for pwCF ≥ 6 years of age harboring at least one F508del allele. However, access to ETI for younger people or those with non-F508del responsive variants has in many instances been obtained through court orders, a well-established but inequitable mechanism for accessing high-cost therapies in the Brazilian health care system.^6^

Documenting the real-world clinical experience with ETI at a referral center for cystic fibrosis patients in Brazil is relevant for several reasons: it illustrates the drug’s effectiveness in a population with distinct genetic and socioeconomic characteristics; it provides evidence to inform national and regional health policy decisions; and it strengthens the global evidence supporting equitable access to HEMT in middle-income settings.^3^ ^-^ ^5^ The objective of the present study was therefore to describe preliminary real-world clinical outcomes associated with ETI in pwCF followed at a pediatric referral center in Brazil, as well as to situate our findings within the broader international evidence. 

## METHODS

This was a cohort study conducted through a review of medical records of pwCF followed at a pediatric referral center in Brazil. This was a retrospective analysis nested within a prospective cohort study. 

Eligibility criteria were as follows: (1) a confirmed diagnosis of cystic fibrosis based on clinical features and CFTR genotyping; (2) initiation of ETI during the study period; and (3) at least six months of follow-up after ETI initiation. The eligibility criteria for ETI within the SUS are being ≥ 6 years of age and having at least one F508del allele.^7^ PwCF who did not meet those criteria-including children in the 2- to 6-year age bracket and individuals carrying non-F508del variants recognized as ETI-responsive by the U.S. Food and Drug Administration and the European Medicines Agency labels-were not eligible for treatment within the SUS and were included if they had obtained access to the drug combination through a court order. PwCF with fewer than six months of follow-up or who were transferred to another center before the first follow-up assessment were excluded. 

Demographic, genetic, and clinical variables were retrospectively extracted from medical records. Key outcomes included FEV_1_, BMI-for-age z-scores (BAZ), weight-for-age z-scores, SCC, liver function tests (including ALT and AST), creatine phosphokinase, microbiological surveillance data, and adverse events. All variables were recorded at baseline (immediately before ETI initiation) and at scheduled follow-up visits at 6-12 months after initiation of ETI. 

Weight and height were measured at each clinical visit with calibrated equipment (Marte Científica & Instrumentação Industrial Ltda., Santa Rita do Sapucaí, Brazil). BAZ and weight-for-age z-scores were calculated in accordance with WHO reference charts, being stratified by biological sex and age. 

Sweat was collected by pilocarpine iontophoresis, and SCC was measured by chloridometry, in accordance with standardized guidelines. 

Spirometry was performed in pwCF aged ≥6 years using calibrated equipment, in accordance with internationally accepted recommendations for pulmonary function testing. Up to November 2025, spirometric assessments were performed using the MS-IOS digital system (Jaeger, Würzburg, Germany). From December 2025 onward, spirometry was performed using the Vyntus™ BODY system (Vyaire Medical Inc., Chicago, IL, USA). All spirometry tests were performed in accordance with the recommendations of the American Thoracic Society and the European Respiratory Society and standardized criteria for technique, acceptability, and reproducibility. Reference equations were selected by the following criteria: 2012 Global Lung Function Initiative values^8^ were applied as the primary reference for all pwCF ≥ 7 years of age; the reference values proposed by Jones et al.^9^ were used for children in the 6- to 6.9-year age bracket because the 2012 Global Lung Function Initiative lower-bound estimates are less well validated in Brazilian children in that age group. The best of the measurements obtained at 6-12 months after ETI initiation was used as the post-treatment assessment for lung function. 

Surveillance sputum or oropharyngeal swab cultures were routinely obtained every three months. Respiratory microbiological surveillance was performed by using routine cultures of respiratory samples collected during outpatient visits or hospitalizations. Specimens included spontaneously expectorated sputum or oropharyngeal swabs. Samples were processed by using conventional microbiological techniques, with inoculation onto selective and nonselective culture media routinely used for detection of cystic fibrosis-associated pathogens. Plates were incubated under appropriate conditions and examined by experienced microbiologists for pathogen identification in accordance with standard laboratory procedures. 

At ETI initiation, pwCF and caregivers were instructed to report adverse events-including skin rash, urticaria/angioedema, nausea, vomiting, and abdominal pain-by telephone or during scheduled consultations. Liver function tests and creatine phosphokinase levels were monitored every three months as part of safety surveillance. 

Data were analyzed with GraphPad Prism, version 8.0 (GraphPad Software, Boston, MA, USA). Categorical variables are described as absolute and relative frequencies. Continuous variables are presented as mean ± standard deviation or median and interquartile range, depending on the normality of the data distribution. Normality was assessed with the Shapiro-Wilk test. Comparisons between baseline and follow-up measurements were performed with the paired Student’s t-test for normally distributed variables or the Wilcoxon signed-rank test for non-normally distributed variables. Differences in categorical variables were assessed with the chi-square test. Correlation analyses were performed by using Spearman’s rank correlation coefficient. A two-tailed value of p < 0.05 was considered significant. 

The present study was approved by the local research ethics committee (Protocol no. CAAE 86300325.2.0000.5269) and was conducted in accordance with the principles of the Declaration of Helsinki and applicable Brazilian regulations governing human research (Brazilian National Health Council Resolution nos. 466/2012 and 510/2016). 

## RESULTS

Between February of 2023 and February of 2026, 113 pwCF initiated treatment with ETI at our center; of those, 108 had at least six months of follow-up and were therefore included in the present analysis. The main reasons for exclusion of 5 individuals were fewer than six months of follow-up (n = 4) and transfer to another center (n = 1). Demographic and baseline clinical characteristics of the 108 pwCF included in the present analysis are summarized in Table 1. Missing data were handled as follows: FEV_1_ was unavailable for 6 of 108 pwCF, all of whom were < 6 years of age at ETI initiation. Repeat sweat chloride data were unavailable for 23 pwCF, and repeat microbiological data were unavailable for 2. 

**Table 1:** Demographic and baseline clinical characteristics of people with cystic fibrosis who initiated treatment with a triple combination of elexacaftor, tezacaftor, and ivacaftor and who had ≥ 6 months of follow-up (N = 108).^a^

Variable	n	Value
Age at ETI initiation, years	108	10.2 ± 3.7
Biological sex	108	
Female	53	49.1%
Male	55	50.9%
CFTR genotype class	108	
F/F (p.Phe508del / p.Phe508del)	50	46.3%
F/non-F (p.Phe508del / other variant)	51	47.2%
Most common second variants (F/non-F)		
3120+1G>A	6	11.8% of F/non-F
G542X	6	11.8% of F/non-F
G85E	5	9.6% of F/non-F
3849+10KbC>T	2	3.9% of F/non-F
A561E	2	3.9% of F/non-F
R1162X	2	3.9% of F/non-F
Other (one occurrence each, n = 28)	28	54.9% of F/non-F
non-F/non-F (two non-F508del variants)*	7	6.5%
Duration of ETI use, months	108	19 [6-51]
Prior CFTR modulator (lumacaftor/Ivacaftor)	108	12 (11.1%)
Baseline BMI-for-age z-score	108	−0.23 (−0.91 to 0.77)
Baseline FEV_1_%	102	80.5 [60-97.2]
Baseline sweat chloride, mmol/L	100	95 [81.5-98]

ETI: elexacaftor/tezacaftor/ivacaftor; CFTR: cystic fibrosis transmembrane conductance regulator; and FEV_1_%: percent predicted FEV_1_. ^a^Data are expressed as n (%), mean ± SD, or median [IQR]. *Two non-F508del variants*: R117H/A559T*; *G85E/Y1092X*; *3120+1G>A/L1077P*; *G85E/S434X*; *1507DEL/N1303K*; *V232D/2148delA*; and/or *G85E/S4X*. Note: Proportions for second variants within the F/non-F group are calculated from the 51 individuals with this genotype class.

ETI was associated with a significant improvement in nutritional status. As can be seen in Figure 1, the median BAZ increased from −0.23 [IQR, −0.91 to 0.77] at baseline to 0.27 [IQR, −0.43 to 1.07] after treatment (p < 0·0001). 

**Figure 1:**
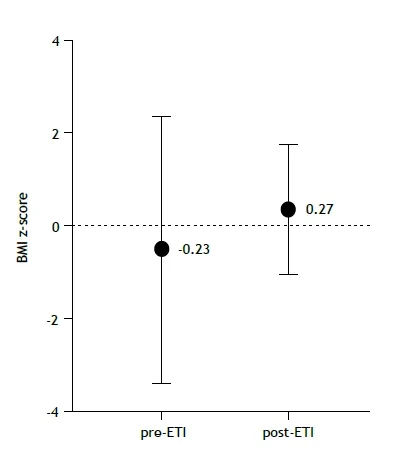
Median BMI-for-age z-scores before and after 6-12 months of elexacaftor/tezacaftor/ivacaftor (ETI) use.

Lung function improved substantially after ETI initiation. Among the 102 pwCF with spirometry available at 6-12 months of follow-up, median ppFEV_1_ increased from 80.5 [IQR, 60-97.25] at baseline to 97.5 [IQR, 81.2-108] after treatment (p < 0.0001), as shown in Figure 2. When we analyzed each person’s individual gain, the mean increase in FEV_1_ percentage points (ppFEV_1_) was 16.9. 

**Figure 2:**
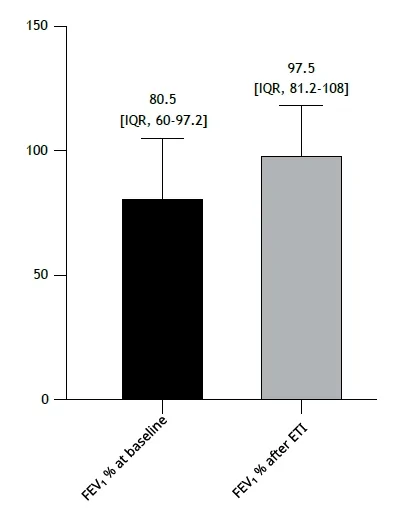
Median percent predicted FEV_1_ (FEV_1_%) and IQR before and after 6-12 months of elexacaftor/tezacaftor/ivacaftor (ETI) therapy.

In the 83 pwCF with repeat sweat testing at 6-12 months after ETI initiation, SCC decreased substantially from a median of 95 mmol/L [IQR, 81.5-98] at baseline to 41 mmol/L [IQR, 31-58] after treatment (p < 0.0001), with the majority of individuals achieving values below the diagnostic threshold of 60 mmol/L (Figure 3). Of the 83 pwCF with repeat sweat testing, 62 (74.7%) achieved SCC < 60 mmol/L and 18 (21.7%) achieved SCC < 30 mmol/L. 

**Figure 3:**
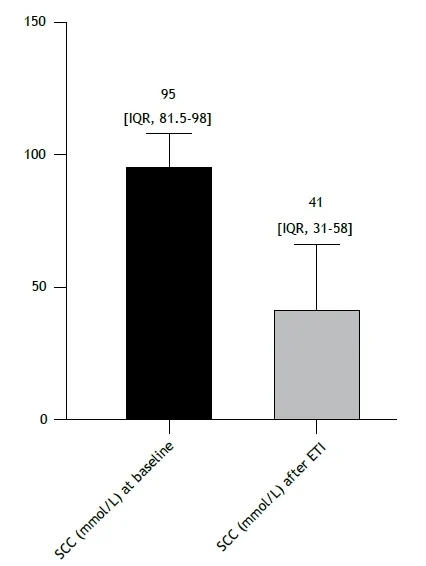
Median sweat chloride concentration (SCC) and IQR before and after elexacaftor/tezacaftor/ivacaftor (ETI) therapy.

There was no significant correlation between the magnitude of SCC reduction and changes in BAZ (Spearman *r* = −0.14; p = 0.24; n = 85) or improvement in ppFEV_1_ (Spearman *r* = −0.01; p = 0.89; n = 79). 

In the year preceding ETI initiation, 59 pwCF (54.6%) had at least one sputum or oropharyngeal swab culture positive for *Pseudomonas aeruginosa*. During the 6-12 months following ETI initiation, the detection rate declined significantly, with only 25 of 106 pwCF (23.6%) having a positive culture (p < 0.0001). Repeat culture data were unavailable for two individuals. 

Six individuals (5.6%) developed skin rash following ETI initiation, which prompted temporary withdrawal of the medication in all cases; ETI was subsequently reintroduced and tolerated without recurrence in the majority. Two individuals had elevated creatine phosphokinase levels, which were related to increased physical activity and which resolved without dose modification. No individual demonstrated a clinically significant elevation in liver enzymes (ALT or AST exceeding three times the upper limit of normal) at any point during follow-up. No permanent discontinuation of ETI was needed. 

## DISCUSSION

In this real-world cohort of pwCF followed at a pediatric referral center in Brazil, ETI therapy was associated with significant improvements in nutritional status, lung function, and CFTR function restoration at 6-12 months after initiation of treatment. The safety profile was favorable, with no cases of serious or irreversible adverse events. These findings are broadly consistent with the results of pivotal clinical trials and post-approval registries from high-income countries and constitute an important contribution to the nascent evidence base on HEMT in Latin America.^1^ ^,^ ^5^ ^,^ ^9^

The present cohort also reflects the remarkable genetic diversity that characterizes the Brazilian cystic fibrosis population. Notably, 6.5% of the study population carried two non-F508del variants, and more than half of those with a heterozygous genotype (F/non-F) harbored a rare or population-specific variant on the second allele-a landscape that stands in stark contrast to the predominantly F508del-homozygous profiles reported in European and North American registries. This diversity, which mirrors the broader admixed genetic background of the Brazilian population, underscores the importance of generating country-specific real-world evidence and reinforces the urgent need to expand ETI eligibility and SUS coverage beyond the F508del restriction, so that all pwCF, regardless of genotype, may have access to the therapeutic benefits of HEMT. 

The significant improvement in BAZ observed in our cohort aligns with the nutritional benefits of ETI documented in pediatric and adult populations worldwide.^9^ CFTR dysfunction impairs exocrine pancreatic function in approximately 85-90% of pwCF,^1^ leading to fat malabsorption, chronic caloric deficit, and growth failure. Additionally, the systemic inflammatory burden imposed by recurrent pulmonary infections substantially increases resting energy expenditure. Longer-term follow-up will be essential to determine whether sustained BAZ gains are achievable at the population level in this context.^4^ ^,^ ^5^

The mean individual FEV_1_ gain of 16.9 percentage points observed in our cohort is comparable to improvements reported in other real-world pediatric series in Brazil (Δ ≈ 11-16pp) and is broadly consistent with findings from pivotal phase III trials, which reported mean ppFEV_1_ gains of 10-14 in adolescents and adults, and up to 10 in children in the 6- to 11-year age bracket.^2^ ^-^ ^5^ Notably, the wide IQRs in ppFEV_1_ at baseline and follow-up reflect the heterogeneity of this real-world cohort, which included pwCF ranging from those with near-normal lung function-in whom ceiling effects may limit measurable gains-to those with advanced bronchiectasis, in whom functional recovery may be attenuated by fixed structural damage. The inclusion of pwCF presenting with non-F508del variants and accessing ETI via legal injunction further contributes to this variability, given that the molecular response to ETI may differ according to the specific variant and its predicted impact on CFTR protein processing. Despite this heterogeneity, the population-level improvement was significant and clinically relevant. Our findings support the concept that real-world pediatric cystic fibrosis populations in Brazil can achieve lung function benefits from ETI comparable to those observed in clinical trials, even in settings with resource constraints.^5^

The pronounced reduction in SCC from a median of 95 mmol/L to 41 mmol/L confirms robust pharmacodynamic activity of ETI in our cohort, with most of the pwCF achieving levels below the 60 mmol/L diagnostic threshold for cystic fibrosis. SCC reflects the direct in vivo effect of ETI on epithelial chloride transport and is among the most sensitive and reproducible biomarkers of CFTR modulation. The magnitude of reduction observed in our series is consistent with data from clinical trials and registry studies, which have documented median SCC reductions of 40-55 mmol/L with ETI across genotype groups.^2^ ^-^ ^4^ The residual SCC values observed in some pwCF likely reflect the incomplete correction of the most severely misprocessed CFTR variants, particularly those associated with minimal residual function. Importantly, SCC reduction was measurable even in pwCF with non-F508del variants, supporting the pharmacological rationale for expanding ETI eligibility beyond the F508del genotype. 

The absence of a significant correlation between SCC reduction and improvements in ppFEV_1_ or BAZ in our cohort is noteworthy and has been reported in several real-world studies.^1^ ^,^ ^5^ This dissociation has at least three plausible explanations. First, SCC reflects a near-instantaneous biochemical response to ETI, whereas clinical outcomes such as lung function and nutritional status integrate decades of disease-related damage that cannot be fully reversed in the short to medium term, regardless of the degree of CFTR restoration. Second, baseline disease severity is an important modifier of clinical response: pwCF with early-stage disease and relatively preserved lung function show proportionally smaller ppFEV_1_ gains despite equivalent or greater CFTR biochemical restoration, giving rise to a ceiling effect that attenuates correlations. Third, the clinical response to ETI in domains such as lung function is modulated by additional factors-including the burden of preexisting bronchiectasis, airway microbiology (particularly the presence of *P*. *aeruginosa* chronic infection), systemic inflammation, adherence to concurrent therapies, and individual pharmacokinetic variability-that are not captured by SCC alone. Taken together, these observations reinforce the view that SCC should be regarded as a sensitive but context-dependent surrogate of CFTR modulation rather than a direct predictor of clinical outcomes in individual pwCF.^1^ ^,^ ^5^

The reduction in *P*. *aeruginosa* detection observed in our cohort after initiation of ETI is consistent with findings reported in real-world studies and registry analyses. In our population, the proportion of individuals with at least one positive respiratory culture for *P*. *aeruginosa* decreased from 54.6% in the year preceding ETI initiation to 23.6% within 6-12 months of therapy. Similar reductions in *Pseudomonas* detection have been reported in observational cohorts following ETI initiation,^10^ ^,^ ^11^ although complete eradication is not universal. Several studies have demonstrated that a substantial proportion of pwCF with established chronic *Pseudomonas* infection remain culture-positive despite clinical improvement and reduced bacterial density in airway samples. Persistent colonization is particularly common among individuals with long-standing infection, older age, and more advanced structural lung disease, suggesting that airway damage and established bacterial biofilms may limit complete microbial clearance even in the setting of highly effective CFTR modulation.^10^

Another important factor influencing the interpretation of microbiological outcomes after ETI therapy is the marked reduction in sputum production observed in many individuals receiving CFTR modulators. Improved mucociliary clearance and reduced airway inflammation frequently lead to decreased availability of sputum samples, which can limit microbiological surveillance of the lower airways. As a result, decreases in culture positivity may partly reflect reduced sampling rather than complete eradication of airway pathogens. This limitation has been acknowledged in several post-ETI microbiological studies and should be considered when interpreting changes in pathogen detection over time.^10^ ^,^ ^11^ Therefore, although our findings support the growing body of evidence demonstrating a significant decline in *P*. *aeruginosa* detection following ETI therapy, persistent infection in a subset of pwCF and the reduced availability of sputum samples remain important considerations in the interpretation of microbiological outcomes. 

The adverse event profile in our cohort was consistent with published data from clinical trials and real-world registries. Skin rash was the most common adverse event (5.6%), and although it required temporary ETI withdrawal in all those affected, all were successfully rechallenged in the majority of cases without recurrence, consistent with the known tolerability of ETI rechallenge following rash resolution. Elevated creatine phosphokinase was observed in two people, both in the context of increased physical activity-a well-recognized phenomenon attributed to the improvement in functional capacity that accompanies ETI-mediated clinical recovery rather than direct drug toxicity. The absence of clinically significant hepatotoxicity in our cohort-defined as ALT or AST elevation exceeding three times the upper limit of normal-is reassuring. This finding is consistent with large phase III trials and long-term extension studies, in which hepatic enzyme elevations of this magnitude were rare and generally transient.^3^ ^,^ ^4^ Importantly, no one in our cohort required permanent discontinuation of ETI, underscoring the overall favorable tolerability of the regimen in a real-world pediatric setting. 

A distinctive feature of our cohort is the inclusion of pwCF who obtained ETI access through court orders-a pathway that has been extensively described in the Brazilian health care literature as a mechanism for individuals to compel the SUS to provide medications not yet formally approved or reimbursed.^8^ The real-world impact of ETI incorporation into the SUS has been striking: data from our own center have shown an 84.8% reduction in cystic fibrosis-related hospitalizations among pwCF who initiated ETI in the 12 months following treatment onset, and no deaths in that period compared with three deaths in the preceding year.^12^ These figures underscore the profound clinical and health system implications of timely and equitable access to HEMT. The 12 pwCF (12.4%) with prior exposure to the dual corrector regimen lumacaftor/ivacaftor represent a subgroup of particular interest, given that prior modulator use may theoretically influence the baseline state of CFTR protein processing and clinical trajectory with ETI, a question that warrants dedicated analysis in future studies. The broad variant landscape of our cohort-encompassing 44 different CFTR variants, including multiple rare and private mutations-reflects the genetic diversity of the Brazilian cystic fibrosis population and highlights the importance of genotype-tailored eligibility criteria for HEMT.^13^ ^,^ ^14^ Individual cases from our center have demonstrated that meaningful structural lung recovery-as documented by chest CT-can occur even in pwCF with non-F508del genotypes after ETI initiation, reinforcing the case for expanded regulatory and SUS coverage.^15^ Future expansion of ETI access to pwCF with additional non-F508del responsive variants, consistent with evolving international guidance and the recent label expansion by the Brazilian Health Regulatory Agency, would be expected to further extend the therapeutic benefit across the Brazilian cystic fibrosis population.^3^ ^,^ ^11^

The present study has several limitations. First, its retrospective single-center design may limit the generalizability of the findings and introduces a potential risk for selection bias. Second, our retrospective analysis relied on data recorded during routine clinical care, and treatment adherence (e.g., medication possession ratios or directly observed dosing), attendance at scheduled follow-up visits, and concomitant therapies that may modulate outcomes-including nutritional supplementation and chronic suppressive antibiotic use-were not systematically recorded and could therefore not be formally analyzed as covariates; although all cystic fibrosis patients were followed at a well-structured referral center with active adherence counseling, unmeasured variability in those factors cannot be excluded and should be prospectively captured in future studies. Third, the absence of a concurrent control group precludes causal inference; however, the consistency of our findings with results from placebo-controlled randomized trials renders a confounded result unlikely. Fourth, microbiological outcomes, quality-of-life data, and pulmonary exacerbation rates-although collected as part of routine care-were not systematically analyzed in this preliminary report and will be addressed in subsequent analyses. Finally, between-center variability in spirometry reference equations, anthropometric methods, and sweat chloride testing protocols may limit direct comparability with data from other countries. 

The present findings underscore the need for a prospective, multicenter national registry of pwCF receiving CFTR modulators in Brazil, which would enable systematic monitoring of long-term effectiveness, safety, and equity of access across different regions and socioeconomic strata. Such a registry would also provide the infrastructure needed to investigate subgroup-specific responses, including those in pwCF with uncommon variants, very young children, and individuals with advanced lung disease. As previously argued by our group, expanding research and access to CFTR modulators for pwCF without the F508del mutation is both a scientific and an ethical imperative-countries with high genetic diversity such as Brazil must invest in real-world studies and advocacy to ensure that all pwCF, regardless of genotype, can benefit from transformative CFTR-targeted therapies.^13^ ^,^ ^14^ Integration with existing international cystic fibrosis registries-such as those of the European Cystic Fibrosis Society and the U.S. Cystic Fibrosis Foundation-would further strengthen the evidence base and facilitate comparative analyses.^3^ ^,^ ^4^

In conclusion, ETI therapy in this pediatric cystic fibrosis cohort in Brazil was associated with significant improvements in nutritional status, lung function, and SCC, with a favorable safety profile, consistent with findings from clinical trials and international real-world studies. These real-world findings support the sustained inclusion of ETI in the Brazilian public health care system and, subject to confirmatory genotype-specific analyses, may inform discussions regarding the expansion of eligibility to additional CFTR variants. The establishment of a systematic national cohort registry to monitor long-term outcomes and inform equitable health policy is warranted. 
